# Bioinspired-Metalloporphyrin Magnetic Nanocomposite as a Reusable Catalyst for Synthesis of Diastereomeric (−)-Isopulegol Epoxide: Anticancer Activity Against Human Osteosarcoma Cells (MG-63)

**DOI:** 10.3390/molecules24010052

**Published:** 2018-12-24

**Authors:** Lucas D. Dias, Ana L. M. Batista de Carvalho, Sara M. A. Pinto, Gilberto L. B. Aquino, Mário J. F. Calvete, Liane M. Rossi, M. P. M. Marques, Mariette M. Pereira

**Affiliations:** 1 Coimbra Chemistry Centre, CQC, Departament of Chemistry, Faculdade de Ciências e Tecnologia, Universidad de Coimbra, 3004–535 Coimbra, Portugal; lucasdanillodias@gmail.com (L.D.D.); smpinto@qui.uc.pt (S.M.A.P.); gilberto.benedito@ueg.br (G.L.B.A.); mcalvete@qui.uc.pt (M.J.F.C.); 2 Unidade de I&D Química-Física Molecular, Department of Chemistry, University of Coimbra, 3004–535 Coimbra, Portugal; almbc@ci.uc.pt; 3 Faculty of Pharmacy, State University of Goiás, 75132-400 Anápolis, Goias, Brazil; 4 Departamento de Química Fundamental, Instituto de Química, Universidade de São Paulo, São Paulo 05508–000, Brazil; lrossi@iq.usp.br; 5 Department of Life Sciences, University of Coimbra, Calçada Martim de Freitas, 3000–456 Coimbra, Portugal

**Keywords:** hybrid magnetic nanoparticles, manganese(III) porphyrin, epoxidation, molecular oxygen, terpene, isopulegol, anticancer activity, human osteosarcoma

## Abstract

In the present study, we developed a green epoxidation approach for the synthesis of the diastereomers of (−)-isopulegol benzyl ether epoxide using molecular oxygen as the oxidant and a hybrid manganese(III)-porphyrin magnetic reusable nanocomposite as the catalyst. High activity, selectivity, and stability were obtained, with up to four recycling cycles without the loss of activity and selectivity for epoxide. The anticancer effect of the newly synthesized isopulegol epoxide diastereomers was evaluated on a human osteosarcoma cell line (MG-63); both diastereomers showed similar in vitro potency. The measured IC_50_ values were significantly lower than those reported for other monoterpene analogues, rendering these epoxide isomers as promising anti-tumor agents against low prognosis osteosarcoma.

## 1. Introduction

The worldwide burden of cancer is increasing, as it is expected to rise to 22 million cases per year within the next two decades. Among the variety of malignant cancer cells, osteosarcoma is one of the most common primary malignant bone cancers to occur in childhood and adolescence. Osteosarcoma has a very poor prognosis for patients with metastatic or recurrent disease (survival <20%) [1]. Therefore, the development of new chemical entities that are both non-toxic and active against cancer cells, particularly regarding osteosarcoma, is a high impact challenge. It is well-known that a wide number of compounds isolated from natural products (e.g., essential oils), such as curcumin [2,3,4,5,6], capsaicin [7,8,9,10,11], and terpenes [12,13,14,15,16] have significant in vitro anticancer effects. Monoterpenes, in particular, which are very abundant in plants, display a wide array of biological functions, comprising chemo-preventive and chemotherapeutic activities, although the molecular mechanisms involved are still poorly understood. Among these, limonene, citronellol [17], terpinen-4-ol [18], (−)-isopulegol, and perillyl alcohol have been reported as very promising antitumor agents, namely towards mammary, colon, lung, liver, and pancreatic carcinomas [19,20,21,22,23,24]. Nevertheless, transposition to in vivo therapeutic efficacy is often limited by transport and biodistribution. However, this can be overcome using strategies that enhance bioavailability and cancer cell-targeting [25,26,27], including the preparation of structural analogues [28,29], particularly epoxides [30,31,32,33,34]. Multiple examples can be found in the literature for epoxide synthesis [35,36], but many of these involve stoichiometric and pollutant oxidants such as *m*-chloroperoxybenzoic acid [37] or iodosylbenzene [38]. In order to solve this environmental challenge, the use of clean catalytic oxidative processes using either H_2_O_2_ [39,40,41] or O_2_ [42,43] as non-pollutant oxidants have been extensively investigated, mostly using metalloporphyrins as catalysts [37,38,44]. This class of bioinspired N-ligands, for instance metalloporphyrins, which allow the selective epoxidation of olefins with multiple scaffolds has, however, an intrinsic instability problem, as it is considered to be one of the main drawbacks for the implementation of these epoxide synthetic processes to the pharmaceutical industrial scale. Nevertheless, strategies to overcome this issue, such as the immobilization of metalloporphyrins onto solid supports, namely polymers [45,46,47], clays [48], silica [49,50], mesoporous materials [51,52], or magnetic nanoparticles [43,53,54], is currently the target of particularly active research, as it is considered one of the most promising approaches.

**Figure 1:**
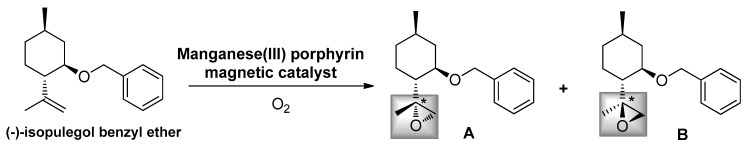
Synthesis of diasteromeric (−)-isopulegol benzyl ether epoxide (A and B), using O_2_ as oxidant and a hybrid manganese (III)-porphyrin magnetic nanocomposite as catalyst.

The aim of the present study is to develop an alternative synthetic strategy for the synthesis of the (−)-isopulegol epoxide diastereomers (A and B) as potential anti-tumor agents against osteosarcoma, through a green epoxidation procedure using molecular oxygen as an oxidant and catalyzed by a hybrid manganese(III)-porphyrin magnetic nanocomposite (Figure 1).

Furthermore, the effect of each epoxide-isopulegol diastereomer on human osteosarcoma cells was evaluated, using two complementary biological screening methods: the MTT (3-(4,5-Dimethylthiazol-2-yl)-2,5-Diphenyltetrazolium Bromide) and the SRB (Sulforhodamine B) colorimetric assays.

## 2. Results and Discussion

### 2.1. Synthesis and Characterization of Hybrid Manganese(III)-Porphyrin Magnetic Catalyst (6)

**Scheme 1:**
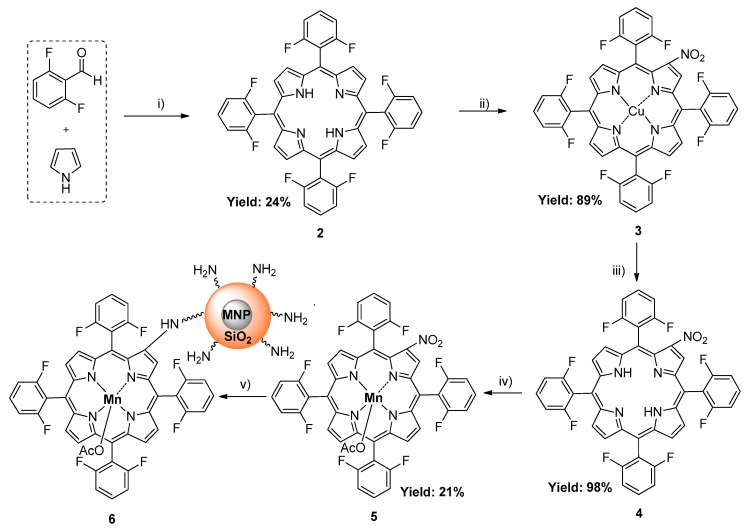
General synthesis of hybrid manganese(III)-porphyrin magnetic nanocomposite(**6**): (**i**) Acetic Acid/Nitrobenzene (3:1); NaY; 150 °C; (**ii**) Cu(NO_2_.3H_2_O; Acetic Anhydride/Acetic Acid (5:1); 60 °C; (**iii**) H_2_SO_4_; 25 °C; (**iv**) Mn(OAc)_2_; DMF; 150 °C; (**v**) CsCO_3_; MNP@SiO_2_-NH_2_ nanoparticles; 85 °C).

The hybrid Mn(III)-porphyrin magnetic nanocomposite (**6**, Scheme 1) was obtained through the covalent linking of β-nitro-metalloporphyrin **5**, Scheme 1 to amine functionalized silica-coated magnetic nanoparticles (MNP@SiO_2_-NH_2_) (Scheme 1). The synthesis of 5,10,15,20-tetrakis(2,6-difluorophenyl)porphyrin (**2**, Scheme 1) was performed by mixing equimolar amounts of pyrrole and 2,6-difluorobenzaldehyde, in a mixture of acetic acid:nitrobenzene (3:1) and Lewis acid NaY (1 g; 0.08 mmol), with further stirring for 2 h, at 150 °C. The product was then purified and isolated in accordance to the NaY reported method [55]. Then, concomitant copper complexation/β-mononitration was carried out by mixing (5,10,15,20-tetrakis(2,6-difluorophenyl)porphyrin (**2**, Scheme 1) with two equivalents of copper nitrate trihydrate, in a mixture of acetic anhydride and acetic acid (5:1), which was stirred for 7 h at 60 °C. The mixture yielded 2-nitro-5,10,15,20-tetrakis (2,6-difluorophenyl) porphyrinatocopper(II) (**3**), Scheme 1 in an 89% yield, after purification and isolation. Subsequently, the demetallation of (**2**, Scheme 1) was performed using sulphuric acid 98% (10 mL), which yielded the desired 2-nitro-5,10,15,20-tetrakis(2,6-difluorophenyl) porphyrin (**4**) (98%). Then, the manganese complex was prepared by mixing 2-nitro-5,10,15,20-tetrakis(2,6-difluorophenyl) porphyrin (**3**, Scheme 1), with an excess of manganese (II) acetate in DMF for 18 h, at 150 °C. Finally, the crude was purified by silica gel column chromatography, using ethyl acetate as an eluent. This yielded 2-nitro-5,10,15,20-tetrakis(2,6-difluorophenyl)porphyrinatomanganese(III) acetate (**5**, Scheme 1) in a 21% yield. Finally, the covalent linking of the β-nitro-metalloporphyrin (**5**, Scheme 1) to the previously prepared MNP@SiO_2_-NH_2_ nanoparticles was carried out using cesium carbonate as a base and DMF as solvent, stirring at 85 °C for 24 h (Scheme 1). Then, the magnetic material was collected by using an external magnetic field and washed (3×) using acetonitrile, ethyl acetate, and dichloromethane. Lastly, the MNP@SiO_2_-NH-TDFPP-Mn(III) (**6**, Scheme 1) was dried under reduced pressure for 48 h, at room temperature (25 °C) and characterized by UV-Vis, thermogravimetry/differential scanning calorimetry (TG-DSC), Fourier transform infrared spectroscopy (FT-IR), and flame atomic absorption (FAAS).

**Figure 2:**
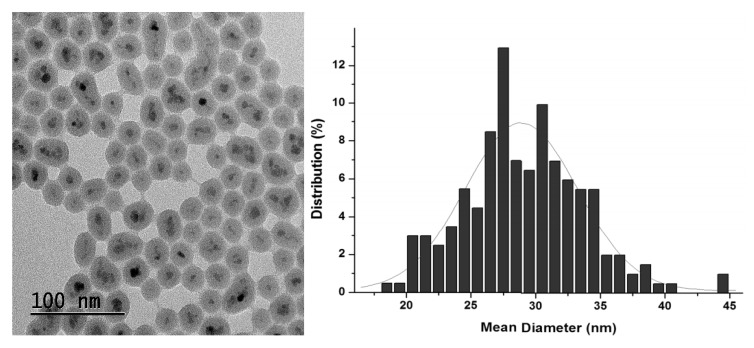
Transmission electron microscopy (TEM) images (**left**) and the corresponding particle size distribution histograms of MNP@SiO_2_ expressed as distribution as function of mean diameter (**right**).

The manganese content of hybrid manganese(III)-porphyrin magnetic nanocomposite (**6**, Scheme 1) was quantified using flame atomic absorption spectroscopy (FAAS), indicating a Mn percentage of 0.9%, which corresponds to 0.99 µmol of porphyrin (**5**, Scheme 1) per gram of nanocomposite (**6**, Scheme 1). Transmission electron microscopy (TEM) images (Figure 2) show that the silica-coated magnetic nanoparticles (MNP@SiO_2_) exhibit a homogeneous spherical silica-coated morphology, with an average diameter of 29 ± 5 nm, whereas magnetic nanoparticle (MNP) has a size of 8 ± 2 nm.

**Figure 3:**
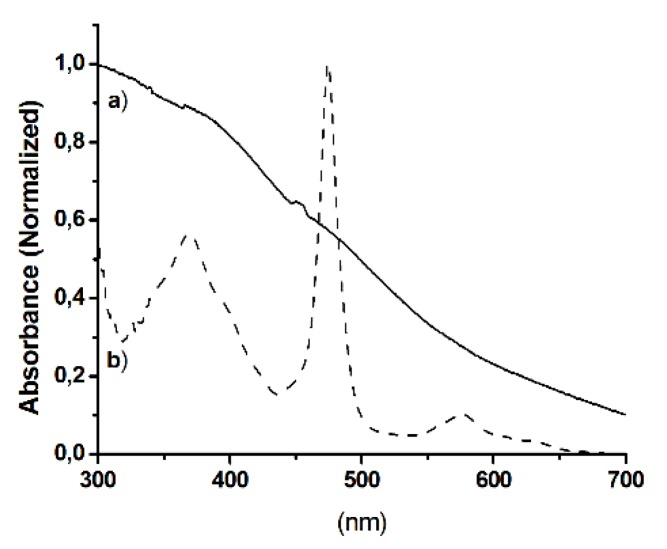
UV-Vis spectra of: (**a**) MNP@SiO_2_-NH-TDFPP-Mn(III), (**6**) in DMF; (**b**) non-immobilized metaloporphyrin (**5**), in CHCl_3_, expressed as normalized absorption as function of wavelength.

The UV–Vis absorption spectrum of (**6**, Scheme 1) displays a typical Soret band at 468 nm, which is in accordance to the Soret band at 475 nm of the non-immobilized metalloporphyrin **5** (Figure 3). This shift can be explained based on the orientation of the nanoparticle molecular dipole with respect to the porphyrin-nanoparticle axis [56].

**Figure 4:**
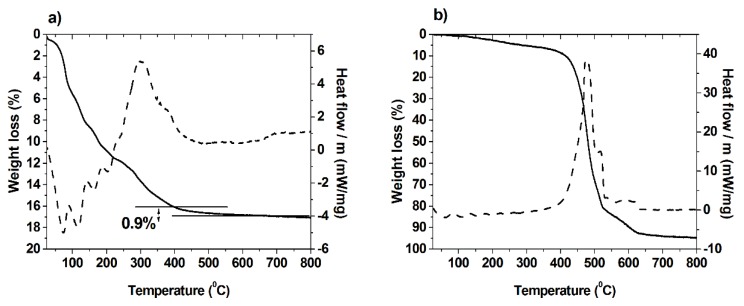
Thermogravimetry/differential scanning calorimetry (TG-DSC) curves (weight loss–solid line; heat flow–dashed line) of: (**a**) MNP@SiO_2_-NH-TDFPP-Mn(III) (**6**); (**b**) β-nitro-metalloporphyrin (**5**). (**a**,**b**) are expressed as weight loss as function of temperature.

The quantification of organic content in the hybrid manganese(III)-porphyrin magnetic catalyst (**6**) was also carried out using thermogravimetry/differential scanning calorimetry (TG-DSC) measurements between 25 °C and 800 °C (Figure 4). Both diagrams show an endothermic peak between 50 °C and 200 °C, attributed to the weight loss of physically adsorbed solvents and water. The TG-DSC of hybrid Mn(III)-porphyrin magnetic catalyst (**6**) shows an exothermic peaks between 200 °C and 800 °C, which can be attributed to the decomposition of organic material, specifically the decomposition of metalloporphyrin (**5**). Thus, metalloporphyrin grafted onto amine silica-coated magnetic nanoparticles (MNP@SiO_2-_NH_2_) was calculated with the TG-DSC of MNP@SiO_2_-NH_2_ and MNP@SiO_2_-NH-TDFPP-Mn(III) (**6**), which resulted in an amount of 0.9% for metalloporphyrin (**5**). This quantification 0.9% using TG-DSC is in agreement with that determined by FAAS.

**Figure 5:**
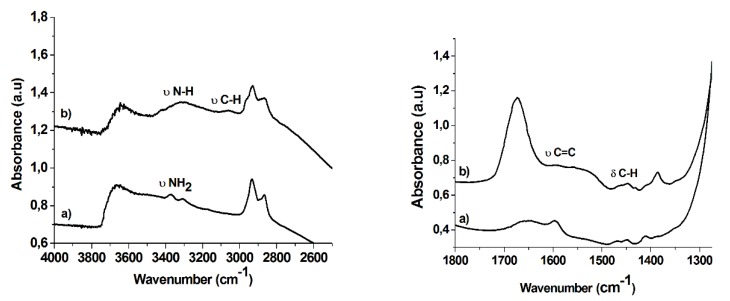
Fourier transform infrared (FTIR) spectra of: (a) MNP@SiO_2_-NH_2_ and (b) MNP@SiO_2_[4-NH-Mn-TDCPP] (**6**), in the regions 4000–2600 (**left**) and 1800–1300 (**right**).

The IR spectrum of MNP@SiO_2_-NH-TDFPP-Mn(III) (**6**) corroborated with the grafting of metalloporphyrin (**5**) onto the functionalized magnetic nanoparticles, especially in comparison with the IR spectrum of MNP@SiO_2_-NH_2_ (Figure 5). In the interval 4000–2600 cm^−1^ (Figure 5, left), both IR spectra show broad bands at 3750–3400 cm^−1^, which is typical of terminal silanol and hydroxyl groups in interaction. In the interval 1300–1700 cm^−1^ (Figure 5, right), the MNP@SiO_2_[4-NH-Mn-TDFPP] (**6**) IR shows several characteristic peaks at: 3300 cm^−1^ (N-H stretching), 3150–3030 cm^−1^ (aromatic C-H stretching), 1625 cm^−1^, 1585 cm^−1^ (aromatic C=C stretching), and 1465 cm^−1^ (C–H bending); these results verify the presence of metalloporphyrin (**5**) in the nanocomposite.

### 2.2. Epoxidation Reaction of (−)-Isopulegol Benzyl Ether (1)

**Scheme 2:**
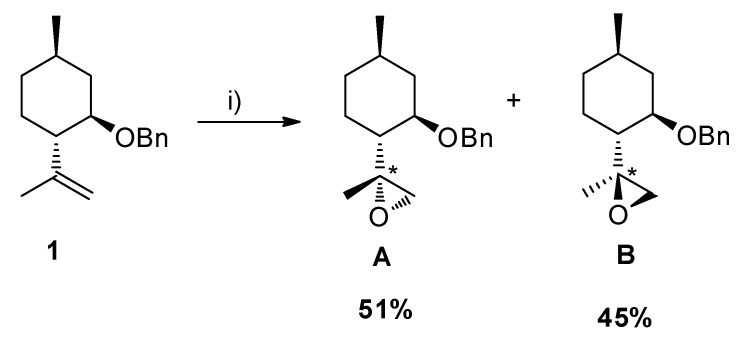
Catalytic epoxidation of (−)-isopulegol benzyl ether (1) catalysed by hybrid manganese(III)-porphyrin magnetic nanocomposite (**6**), yielding diasteroisomers **A** (51%) and **B** (45%). Reaction conditions: catalyst 6; isobutyraldehyde; butyronitrile; O_2_ (5 bar); 25 °C; 6 h.

Considering the high interest in promoting the development of new and more effective osteosarcoma drugs using an efficient and eco-friendly approach, the new hybrid manganese(III)-porphyrin magnetic catalyst (**6**) was used to promote the catalytic epoxidation of (−)-isopulegol benzyl ether (**1**) (^1^H NMR, ^13^C NMR and MS spectrum are shown in Figures S1–S3). This was accomplished using molecular oxygen as a green oxidant and isobutyraldehyde as a co-reductant. In a typical procedure, (−)-isopulegol benzyl ether (**1**) (122 mg, 0.5 mmol), hybrid manganese(III)-porphyrin magnetic catalyst (**6**) (4.7 × 10^−5^ mmol), isobutyraldehyde (0.25 mL, 2.5 mmol), and chlorobenzene (22 mg; 1.95 × 10^−1^ mmol) was the internal standard in butyronitrile and added to an autoclave. Then, the reactor was pressurized with molecular oxygen (5 bar), and kept at room temperature (25 °C), under stirring for 6 h (Scheme 2).

**Figure 6:**
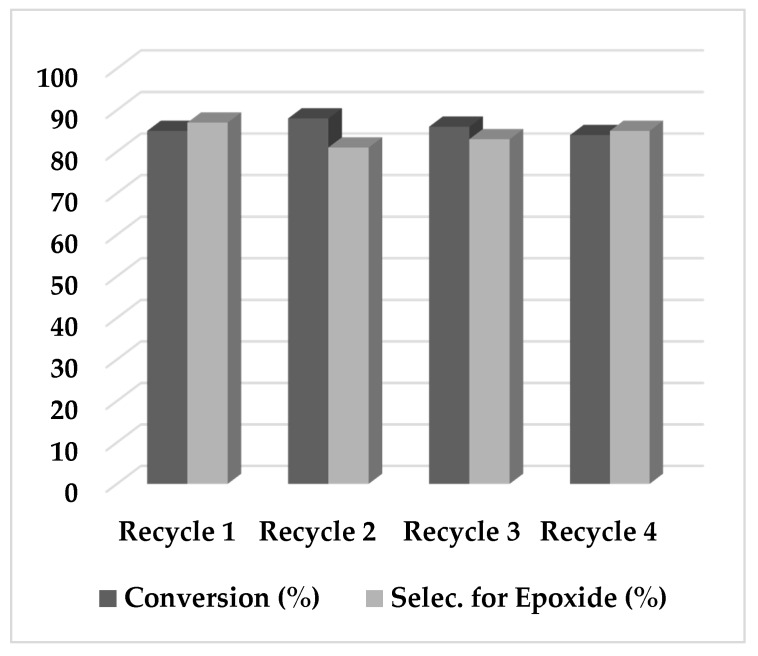
Recycling experiments of MNP@SiO_2_-NH-TDFPP-Mn(III) (**6**) in (−)-isopulegol benzyl ether (**1**) epoxidation. Dark grey refers to conversion (%) and light grey refers to selectivity for epoxide (%).

At the end of the reaction, the hybrid manganese(III)-porphyrin magnetic catalyst (**6**) was recovered using an external magnet field and reused in four consecutive cycles. Between each cycle, the solid material was washed with CH_3_CN (3x) and subsequent solvent removal, then dried under vacuum at 60 °C for 12 h. The hybrid manganese(III)-porphyrin magnetic catalyst (**6**) was then reused for the next run (Figure 6).

**Figure 7:**
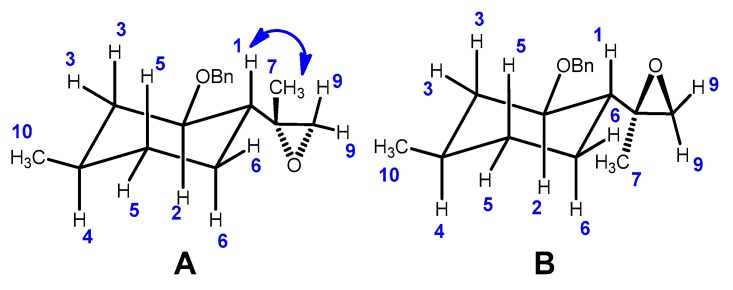
Structures of diastereomers **A** and **B** of (−)-isopulegol benzyl ether epoxide and the NOE cross-peaks. NOESY spectra of diastereoisomer **A** shows an interaction between H1-H7(CH_3_), indicating a proximity between these protons. For the diastereoisomer **B**, the NOE H1-H7 (CH_3_) was not observed.

The hybrid manganese(III)-porphyrin magnetic nanocomposite (**6**) exhibited an excellent recycling capacity. After four consecutive runs, no loss of activity or selectivity for (−)-isopulegol benzyl ether epoxides **A** and **B** (Figure 7) was observed. Furthermore, other than the high activity and selectivity, it is worth noting the great stability of the manganese(III) porphyrin magnetic material, MNP@SiO_2_-NH-TDFPP-Mn(III) (**6**), as no catalyst leaching was detected upon four cycles, which was herein demonstrated using UV-Vis analysis of the reaction’s crude. These stabilizing effects of the metalloporphyrins onto magnetic nanomaterial support may be attributed to the formation of catalytically inactive oxo-dimeric metalloporphyrin species [57].

The crude mixture was washed with a sodium bicarbonate saturated solution and water, where the (−)-isopulegol benzyl ether epoxides **A** and **B** were isolated from the organic phase by simple destillation. Conversion and selectivity were determined using GC (Gas Chromatography), based on peak areas and chlorobenzene as the internal standard. Finally, diastereoisomers **A** and **B** of (−)-isopulegol benzyl epoxide were separated by silica gel column chromatography, using a mixture of ethyl acetate: *n*-hexane (1:4) as an eluent, which yielded a pair of both diastereomers **A** and **B** (Scheme 2) with a combined yield of 51% and 45%, respectively. The diastereomers **A** and **B** were characterized by ^1^H, ^13^C NMR and 2D NMR (Nuclear Magnetic Resonance) techniques (COSY (Correlated Spectroscopy), NOESY (Nuclear Overhauser Effect SpectroscopY), HMQC (Heteronuclear Multiple-Quantum Correlation),and HMBC (Heteronuclear Multiple Bond Correlation). The stereochemistry of the epoxides ring was assigned based on NOE (Nuclear Overhauser Effect) between relevant protons (H1–H7(CH_3_)) (see Figures S4–S15).

NOESY spectra of diastereoisomer **A** shows an interaction between H1-H7(CH_3_), indicating a proximity between these protons (Figure S7). On the other hand, for the diastereoisomer **B**, the NOE H1–H7 (CH_3_) was not observed (Figure S13), which allowed us to propose its stereochemistry.

### 2.3. Biological Evaluation

The anti-tumor effect of the two (−)-isopulegol epoxide diastereomers currently synthesized was assessed through two complementary colorimetric methods: the MTT assay, which assesses cell viability, and the SRB technique, which measures cell density and thus reveals cell proliferation.

**Figure 8:**
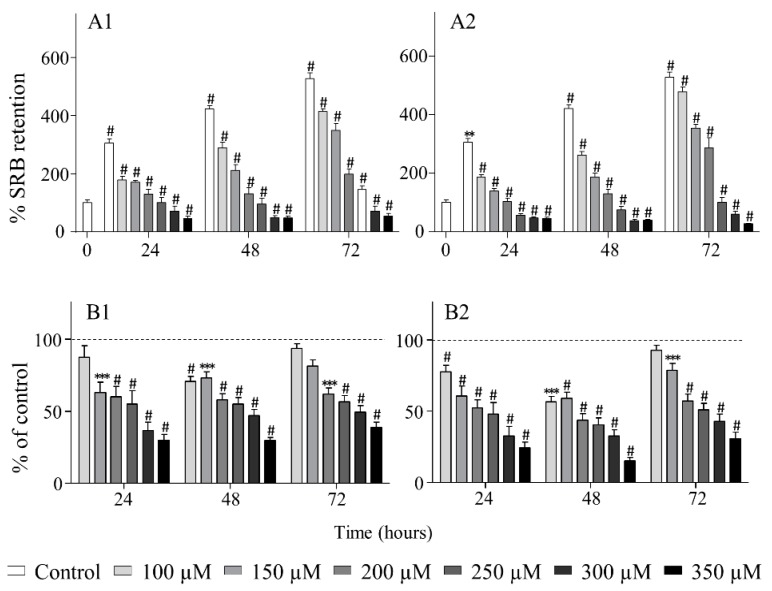
Dose and time-dependent response plots of the effect of epoxide **A** (**A1**,**B1**) and epoxide **B** (**A2**,**B2**) on cell viability, for the MG-63 cell line for dosages between 100 and 350 µM. Results obtained by the sulforhodamine B (SRB) (**A1**,**A2**) and 3-[4,5-dimethylthiazol-2-yl]-2,5 diphenyl tetrazolium bromide (MTT) (**B1**,**B2**) assays, are represented as a percentage of the control (cells treated with the dimethyl sulfoxide (DMSO) vehicle). The data are standard error of the mean ± SEM of the values obtained for 3 independent experiments (*n* = 3), carried out in triplicate. ** *p* < 0.01, *** *p* < 0.001, # *p* < 0.0001 versus control cultures.

Both diastereomers proved to be moderately active towards the human osteosarcoma MG-63 cell line regarding their impact on cell-growth inhibition and on viability, with IC_50_ values (at 48 h) of 114.8 µM (SRB)/249.8 µM (MTT) and 104.0 µM (SRB)/158.2 µM (MTT), respectively, for isomers **A** and **B** (Figure 8). These values are significantly lower than those published for similar compounds, namely for limonene (extracted from mandarin oil) against human lung adenocarcinoma (A549) (Sigma-Aldrich catalogue nr 86012804, Lisbon, Portugal and hepatocarcinoma (HepG2) cells (ATCC^®^ catalogue nr HB-8065™, LGC Standards S.L.U. Barcelona, Spain), where IC_50_ was equal to 586 and 889 µM [23], respectively. In addition, no significant variation was observed between the antineoplastic activity of each of the two diasteromers. This is in agreement with previous studies on the stereochemistry dependence of the anticancer potency, namely for perillyl alcohol [20], tubulysins [58], or enigmol [59]. In fact, although the absolute stereochemistry of a compound may affect its biological activity (with several reported cases), the most relevant structure-activity relationships (SAR´s) rely on the specific structural scaffold and functional groups (pharmacophores) of the molecule.

Despite the scarce knowledge on the precise molecular mechanisms underlying the anticancer activity of monoterpenes, some reported studies reveal an impact on key regulatory signal transduction proteins, particularly from the Ras family, which regulated fundamental processes such as cell growth, differentiation, and survival. Actually, reduced Ras levels (due to decreased up-regulation) were measured in distinct cell lines in the presence of monoterpenes [20], with a strong effect on protein synthesis and degradation and therefore on cell proliferation and viability.

## 3. Materials and Methods

### 3.1. Materials

All chemicals and solvents were acquired from Sigma-Aldrich (Lisbon, Portugal) and Fluorochem (Derbyshire, UK) and used without further purifications. Thin layer chromatography (TLC) was performed on silica gel 60 TLC-plates with P254 indicator (Fluorochem, Derbyshire, UK). Gas chromatography analysis was performed using an HP-Agilent 6890 and HP-G11800A chromatographs (Agilent, Santa Clara, CA, USA), equipped with an ionization detector (FID) and a non-polar HP-5 column (5% diphenyl, 95% dimethylpolysiloxane, 30 m × 0.32 mm). GC-MS (gas chromatography-mass spectrometry) analysis were performed in a 5973 MSD spectrometer (Hewlett-Packard, Palo Alto, CA, USA), using EI (70 eV), coupled to a Hewlett-Packard Agilent 6890 chromatograph (Hewlett-Packard, Palo Alto, CA, USA) (HP-5 MS column − 30 m × 0.25 mm × 0.25 μm). The GC analysis was performed under the following conditions: initial temperature 373 K (100 °C), rate 333 K min^−1^, final temperature 553 K (280 °C), and gas flow 1.63 mL min ^−1^. The MALDI-TOF MS data were carried out using a Bruker Daltonics flex Analysis apparatus (Bruker Spain, Madrid, Spain). UV-Vis absorption spectra were acquired using a Hitachi U-2010 (Hitachi, Terrasa, Spain). A Cary 5000 UV-Vis-NIR spectrophotometer (Agilent, Santa Clara, CA, USA), equipped with an integrating sphere, was used for solid samples. TEM analyses were performed using a Philips CM 200, operating at an accelerating voltage of 200 kV and the samples were prepared in isopropyl alcohol on a holey carbon-covered copper grids. Size distributions of the nanocomposite was determined using manual analysis of enlarged images with Imagetool sware Version 3.0, (Developed by Bruce McArthur, Digital Liquid Multimedia) in order to get a statistical size distribution and a mean diameter by curve fitting using Gaussian function. The manganese content determination in nanocomposite was performed using FAAS on an atomic absorption spectrophotometer Shimadzu AA-6300 (Shimadzu, Milton Keynes UK), with quartz cells. Thermogravimetric analyses were performed on a TG-DSC Setsys Evo16 instrument (Setaram, Caluire, France), with a heating rate of 10 K min^−1^ up to 800 °C. The infrared spectra were obtained (with 64 scans) on a FTIR Nexus 670 Nicolet spectrometer (Thermo Scientific, Waltham, ME, USA), heating the sample from room temperature up to 150 °C before spectral acquisition in an IR glass cell, for 1.5 h under vacuum. ^1^H, ^13^C, COSY (Correlated Spectroscopy), NOESY (Nuclear Overhauser Effect Spectroscopy), HMBC (Heteronuclear Multiple Bond Correlation), and HMQC (Heteronuclear Multiple-Quantum Correlation) spectra were obtained in CDCl_3_ in Bruker Avance III and Bruker DRX-400 spectrometers (Bruker Spain, Madrid, Spain), both operating at 400.13 MHz for ^1^H and 100.61 MHz for ^13^C. Chemical shifts are expressed in ppm, relatively to an internal standard of TMS.

### 3.2. Synthesis of (−)-isopulegol benzyl ether (1)

(−)-Isopulegol (1.0 g, 6.5 mmol) and NaH (0.192 g, 8 mmol) were added to dry THF (30 mL) and kept under stirring, at room temperature (25 °C). Then, benzyl bromide (1.36 g, 8 mmol) was added dropwise for 30 min and the reaction mixture was kept under stirring, at 50 °C, for 12h. The reaction crude was extracted with ethyl ether (50 mL) and dried using anhydrous sodium sulfate. The solvent was then evaporated under reduced pressure. After purification via silica gel column chromatography, we used a solvent mixture of *n*-hexane:ethyl acetate (9:1) as the eluent, the product (−)-isopulegol benzyl ether (**1**) was isolated in 82 % yield (1.3 g, 5.3 mmol).

^1^H-NMR (CDCl_3_, 400 MHz) δ H (ppm): 7.32–7.19 (m, 5H, HAr), 4.79–4.80 (m, 2H, H9), 4.59 (d, *J* = 16.0 Hz, 1H, H11), 4.41 (d, *J* = 16.0 Hz, 1H, H11), 3.28 (ddd, *J1* = 6.0 Hz, *J2* = 6.0 Hz, *J3* = 6.0 Hz, 1H, H2), 2.18–2.03 (m, 2H, CH), 1.68–1.67 (m, 3H, CH_3_-7), 1.65–1.60 (m, 2H, CH), 1.41–1.25 (m, 2H, CH), 0.93 (s, *J* = 9.0 Hz, 3H, CH_3_-5), 0.99–0.90 (m, 2H, CH). ^13^C-NMR (101 MHz, CDCl_3_) δ C (ppm): 20.1 (C-7), 22.20 (C-10), 31.1 (C-6), 31.6 (C-4), 34.5 (C-5), 40.3 (C-3), 51.8 (C-1), 70.4 (C-11), 79.2 (C-2), 111.1 (C-8), 127.3 (CAr.), 127.6 (CAr), 128.2 (CAr), 139.2 (CAr), 147.9 (C-9). MS (EI) *m*/*z*: 244.1, 229.0, 153.1, 138.1, 123.1, 91.1, 69.1, 41.1.

### 3.3. Synthesis of the Catalyst

#### 3.3.1. 5,10,15,20-tetrakis(2,6-difluorophenyl)porphyrin (2)

This halogenated porphyrin was prepared using reacting pyrrole with the 2,6-difluoro benzaldehyde in equimolar amounts, in a nitrobenzene/acetic acid mixture, according to reported methods [55,60]; characterization data is in agreement with what is previously reported [61].

#### 3.3.2. 2-Nitro-5,10,15,20-tetrakis(2,6-difluorophenyl)porphyrinatocopper(II) (3)

Copper(II) nitrate trihydrate (0.32 g, 1.32 mmol) dissolved in acetic anhydride/acetic acid mixture (5:1) was added to a chloroform solution (500 mL) of 5,10,15,20-tetrakis(2,6-difluorophenyl) porphyrin (**1**) (500 mg, 0.66 mmol); the final solution was stirred for 7 h, at 60 °C. Then, the solvents were evaporated under reduced pressure, washed using a saturated solution of sodium hydrogen carbonate (3×) and water (3×), dried with anhydrous sodium sulfate, and filtered. Finally, the crude was purified using a silica gel column chromatography, with dichloromethane:*n*-hexane (1:1) as the eluent. As a result, 2-Nitro-5,10,15,20-tetrakis(2,6-difluorophenyl) porphyrinatocopper(II) (**3**) was obtained with a 88 % yield (506 mg, 0.58 mmol).

HRMS (MALDI-TOF): Calcd. for C_44_H_19_F_8_N_5_O_2_Cu 864.070; Found: 864.070. UV-vis (toluene): λ_máx_, nm (ε, M^−1^·cm^−1^): 424 (1.6 × 10^5^), 547 (1.2 × 10^4^), 589 (7.1 × 10^3^). EA: Anal. Calcd. for C_44_H_19_F_8_N_5_O_2_Cu: C: 61.07; H, 2.24; N, 8.07. Found: C, 61.08; H, 2.21; N, 8.09.

#### 3.3.3. 2-Nitro-5,10,15,20-tetrakis(2,6-difluorophenyl)porphyrin (4)

In a solution of 2-nitro-5,10,15,20-tetrakis(2,6-difluorophenyl)porphyrinatocopper(II) (**2**) (500 mg, 0.58 mmol) in chloroform (200 mL), sulfuric acid 98% (10 mL) was slowly added, and the reaction mixture was stirred for 1 h at room temperature (25 °C). Then, the reaction was carefully neutralized using a saturated solution of sodium bicarbonate (5×) and water (3×), dried with using with anhydrous sodium sulfate, filtered, and evaporated with the solvent. The crude was purified using a silica gel column chromatography, with dichloromethane:*n*-hexane (2:1) as the eluent. 2-Nitro-5,10,15,20-tetrakis(2,6-difluorophenyl)porphyrin (**4**) was obtained in 98% yield (455 mg, 0.44 mmol).

^1^H-NMR (CDCl_3_, 400 MHz) δ H (ppm): 9.12 (s, 1H, β-H); 8.97–8.89 (m, 4H, β-H); 8.76 (sl, 2H, β-H); 7.86-7.76 (m, 4H, Ar-H); 7.44–7.27 (m, 8H, Ar-H); -2.67 (s, 2H, NH). HRMS (MALDI-TOF): calcd. for C_44_H_21_F_8_N_5_O_2_ 803.160; Found: 803.186.UV-vis (toluene): λ_máx_, nm (ε, M^−1^·cm^−1^): 426 (1.5 × 10^5^), 520 (1.1 × 10^4^), 556 (3.6 × 10^3^), 597 (4.3 × 10^3^), 655 (1.9 × 10^3^).

#### 3.3.4. 2-Nitro-5,10,15,20-tetrakis(2,6-difluorophenyl)porphyrinatomanganese(III) acetate (5)

2-Nitro-5,10,15,20-tetrakis(2,6-difluorophenyl)porphyrin (**3**) (300 mg, 0.37 mmol) was dissolved in DMF (50 mL) and manganese(II) acetate tetrahydrate (910 mg, 3.7 mmol) was added. Then, the reaction was stirred at 150 °C for 18 h. The solvent was evaporated under reduced pressure, washed with water (3×), and dried over anhydrous sodium sulfate. Finally, the crude reaction was purified using a silica gel column chromatography, with ethyl acetate as the eluent. This yielded 2-nitro-5,10,15,20-tetrakis(2,6-difluorophenyl)porphyrinatomanganese(III) acetate (**5**) in 21% (210 mg; 0.24 mmol).

HRMS Matrix-assisted laser desorption/ionization—Time of flight (MALDI-TOF): calcd. for C_46_H_22_F_8_N_5_O_4_Mn 915.090; found: 858.552 [M-CH_3_COO + H]^+^.

UV-vis (toluene): λ_máx_, nm (*ε*, M^−1^.cm^−1^): 368 (4.3 × 10^4^), 477 (7.2 × 10^4^), 580 (7.9 × 10^3^), 616 (3.5 × 10^3^).

EA: Anal. Calcd. for C_46_H_22_F_8_N_5_O_4_Mn: C, 63.46; H, 2.66; N, 6.44. Found: C, 63.32; H, 2.58; N, 6.49.

#### 3.3.5. Synthesis of Magnetic Nanoparticles (MNP)

Magnetite nanoparticles were prepared in the co-precipitation method, using Fe^2+^/Fe^3+^, according to a procedure previously reported [62].

#### 3.3.6. Synthesis of Silica-coated Magnetic Nanoparticles (MNP@SiO_2_)

The silica coating magnetic nanoparticles were obtained using a reverse microemulsion process, according to a previously reported procedure [63].

#### 3.3.7. Synthesis of Amine Functionalized Silica-coated Nanoparticles (MNP@SiO_2_-NH_2_)

MNP@SiO_2_ (1 g) and 3-aminopropyltrietoxysilane (APTES) (0.5 mL; 2.13 mmol) were added to dry toluene; the reaction mixture was kept under stirring for 2 h at 100 °C. At the end, the amine functionalized silica-coated nanoparticles (MNP@SiO_2_-NH_2_) was washed with toluene (3×), separated by centrifugation, and dried at 100 °C for 20 h.

#### 3.3.8. Synthesis of Hybrid Manganese(III)-porphyrin Magnetic Catalyst (**6**)

MNP@SiO_2_-NH_2_ (1 g) and cesium carbonate (340 mg; 1.0 mmol) were added to a round-flask containing a solution of (**5**) (250 mg; 0.29 mmol) in dry 1,2-dimethoxyformide (40 mL). The reaction mixture was put under stirring at 85 °C for 24 h. Then, the magnetic material was collected using an external magnetic field and washed (3×) using acetonitrile, ethyl acetate, and dichloromethane. Finally, the MNP@SiO_2_-NH-TDFPP-Mn(III) (**6**) was dried under reduced pressure for 48 h, at room temperature (25 °C), and characterized by UV-Vis, TG-DSC, FT-IR, and FAAS.

### 3.4. General Procedure for Catalytic Epoxidation of (−)-Isopulegol Benzyl with O_2_ and Scale-Up

An autoclave was charged with the (−)-isopulegol benzyl ether (152 mg; 0.62 mmol) and hybrid manganese(III)-porphyrin magnetic catalyst (**6**) (4.7 × 10^−5^ mmol), butyronitrile (2 mL), isobutyraldehyde (0.25 mL; 2.5 mmol), and chlorobenzene (22 mg; 1.95 × 10^−1^ mmol) as the inert internal standard. The autoclave was pressurised with O_2_ (5 bar) and the mixture reaction was conducted under stirring, at room temperature, 25 °C, for 6 h. The crude mixture was washed with sodium bicarbonate saturated solution (50 mL) and water. The (−)-isopulegol benzyl ether epoxide was then isolated from the organic phase by simple distillation. Conversion and selectivity were determined by GC, based on peak areas, using chlorobenzene as internal standard. Diastereoisomers of (−)-isopulegol benzyl ether epoxide were purified using a silica gel column chromatography, with a mixture of ethyl acetate:*n*-hexane (1:4) as the eluent, yielding 79% of isolated product with diastereosiomers **A** and **B** in 51% (66 mg; 0.26 mmol) and 45% (59 mg; 0.23 mmol), respectively.

The scale-up experiment was performed using (−)-isopulegol benzyl ether (**1**) (0.982 g, 4.02 mmol), isobutyraldehyde (1.168 g, 16.2 mmol), hybrid manganese(III)-porphyrin magnetic catalyst (**6**) (3.04 × 10^−4^ mmol), and butyronitrile (15 mL) as the solvent. Finally, a sodium bicarbonate saturated solution (50 mL) was added to the crude mixture, leading to the formation of two phases. The target epoxide was then isolated in 76% yield (3.05 mmol) from the organic phase by simple distillation. The catalyst (**6**) was steadily separated from the reaction mixture, using an external magnetic stirrer bar, followed by decantation of the liquid. Then, the solid material was washed with CH_3_CN (15 mL), dried under vacuum at 60 °C for 12 h, and the solid was kept under the vacuum and inert atmosphere for reuse.

*(−)-Isopulegol benzyl ether epoxide–diastereoisomer***A**: ^1^H-NMR (CDCl_3_, 400 MHz) δ H (ppm): 7.36–7.25 (m, 5H, HAr), 4.68 (d, *J* = 12.0 Hz, 1H, H11), 4.40 (d, *J* = 12.0 Hz, 1H, H11), 3.26 (ddd, *J1* = 4.0 Hz, *J2* = 4.0 Hz, *J3* = 4.0 Hz, 1H, H2), 2.76 (d, *J* = 8.0 Hz, 1H, H9), 2.68 (d, *J* = 4.0 Hz, 1H, H9), 2.24–2.19 (m, 1H, H3), 1.90–1.86 (m, 1H, H6), 1.71–1.66 (m, 1H, H5), 1.43–1.39 (m, 1H, H6), 1.31–1.24 (m, 1H, H4), 1.13 (s, 3H, CH_3_-7), 1.11–1.08 (m, 1H, H1), 0.95 (d, *J* = 8.0 Hz, 3H, CH_3_-10), 0.92–0.86 (m, 2H, H3, H5); ^13^C-NMR (101 MHz, CDCl_3_) δ C (ppm): 16.7 (C-7), 22.3 (C-10), 26.4 (C-6), 31.5 (C-4), 33.9 (C-5), 39.9 (C-3), 50.8 (C-1), 56.8 (C-9), 57.9 (C-8), 70.2 (C-11), 78.8 (C-2), 127.6 (C-Ar), 128.4 (C-Ar), 139.0 (C-Ar). HRMS (ESI-FIA-TOF): Calcd for C_17_H_24_O_2_ [M + Na^+^] 283.1673; Found *m*/*z* = 283.1669.

*(−)-Isopulegol benzyl ether epoxide–diastereoisomer***B**: ^1^H-NMR (CDCl_3_, 400 MHz) δ H (ppm): 7.42–7.40 (m, 2H, *HAr*), 7.35–7.31 (m, 2H, *HAr*), 7.26–7.23 (m, 1H, *HAr*), 4.72 (d, *J* = 12.0 Hz, 1H, *H11*), 4.44 (d, *J* = 12.0 Hz, 1H, *H11*), 3.32–3.26 (m, 1H, *H2*), 2.51–2.48 (m, 2H, *H9*), 2.23–2.20 (m, 1H, *H3*), 1.75–1.70 (m, 1H, *H6*), 1.69–1.64 (m, 1H, *H5*), 1.45–1.35 (m, 1H, *H4*), 1.34–1.30 (m, 1H, *H1*), 1.25 (s, 3H, *H7*), 1.21–1.11 (m, 1H, *H6*), 0.95 (d, *J* = 6Hz, 3H, *H10*), 0.92–0.85 (m, 2H, *H3* and *H5*). ^13^C-NMR (101 MHz, CDCl_3_) δ C (ppm): 18.9 (C-7), 22.2 (C-10), 27.8 (C-4), 31.3 (C-6), 34.2 (C-5), 39.6 (C-3), 49.2 (C-1), 52.0 (C-9), 58.3 (C-8), 69.7 (C-11), 78.3 (C-2), 127.4 (C-Ar), 127.7 (C-Ar), 128.3 (C-Ar), 138.9 (C-Ar). HRMS (ESI-FIA-TOF): Calcd for C_17_H_24_O_2_ [M+Na^+^] 283.1673; Found *m*/*z* = 283.1669.

### 3.5. Catalyst Recycling

The catalyst (**6**) was steadily separated from the reaction mixture, using an external magnetic stirrer bar, followed by the decantation of the liquid. Then, the solid material was washed with CH_3_CN (2 mL), dried under vacuum at 60 °C for 12 h and reused in the next run.

### 3.6. Biological Evaluation

#### 3.6.1. Cell Culture

The human MG-63 osteosarcoma cell line was obtained from HPA Culture Collections and supplied by Sigma, Portugal. Cells were grown in monolayer in a MEM culture medium, supplemented with 10% (*v*/*v*) heat-inactivated FBS, 1% (*v*/*v*) sodium pyruvate, 1% (*v*/*v*) Non-Essential Amino Acids (NEAA), and 1% (*v*/*v*) penicillin/streptomycin. Cells were maintained at 37 °C under a humidified atmosphere with 5% CO_2_. Cells were subcultured at 80% confluence, using 0.05% trypsin-EDTA (1×) in PBS.

#### 3.6.2. Evaluation of Cytotoxic and Antiproliferative Effects

MG-63 cell cultures were established in 24-well plates (1 mL/well) at a density of 3 × 10^4^ cells/cm^2^ and were allowed to attach for about 24 h. Triplicates were treated for different incubation periods (3 independent experiments) with each of the two diastereomers (**A** and **B**) of (−)-isopulegol benzyl epoxide at concentrations from 350 to 100 μM. Both epoxides were solubilised in DMSO (concentration never exceeding 0.1% (*v*/*v*) in the cell cultures). Cytotoxicity and cell density were evaluated at 24, 48, and 72 h by the MTT [64] and SRB [65] colorimetric assays, respectively. Untreated cell cultures were also analysed, at the same time points, and DMSO was used as the control. The results were expressed as the mean ± SEM value. Statistical analysis was carried out using One-Way ANOVA, followed by the post hoc Turkey’s and Dunnett’s multi-comparison tests for the SRB and MTT results, respectively. The differences were considered significant for *p* < 0.05.

## 4. Conclusions

A hybrid manganese(III)-porphyrin magnetic nanocomposite was prepared and fully characterized by TEM, UV-vis, FAAS, TG-DSC, and FTIR, showing the effective grafting of the manganese(III)-porphyrin (**5**) onto the amine silica-coated magnetic nanoparticles. The nanomaterial was applied as catalyst in the selective epoxidation of (−)-isopulegol benzyl ether (**1**), using molecular oxygen as a green oxidant and isobutyraldehyde as co-reductant. This application aimed to obtain the diastereoisomers of the (−)-isopulegol benzyl epoxide (**A** and **B**) as a potential anti-cancer agent. The developed epoxidation system presented high activity, selectivity, and stability, for up to four recycling cycles.

The two isopulegol epoxide diastereomeric analogues currently studied have showed to be moderately active towards the human osteosarcoma cell line screened (MG-63), both regarding their impact on cell-growth and on viability. Indeed, the IC_50_ obtained between 100 and 250 µM, are quite lower than those previously reported for similar monoterpenes towards different cancer cell lines. In addition, the stereochemical characteristics of the presently tested epoxide was found to have a minor effect on its antitumor activity, which is primarily attributed to structural features rather than to optical properties. Although still ill-understood, a suggested mechanism for this biological activity is a regulatory role related to signal transduction proteins (mainly Ras), that directly affects cell growth and differentiation. These epoxide isomers are promising agents against human osteosarcoma, already in sole administration. Furthermore, combined therapeutic schemes with conventional drugs (e.g., Doxorubicin or Methotrexate) are envisaged, as a way to increase cancer cell sensitization and enhance therapeutic efficacy.

## Supplementary Materials

The following are available online at http://www.mdpi.com/1420-3049/24/1/52/s1, Figure S1: ^1^H NMR spectrum of (–)-isopulegol benzyl ether (1); Figure S2: ^13^C NMR spectrum of (–)-isopulegol benzyl ether (1); Figure S3: MS spectrum of (–)-isopulegol benzyl ether (1); Figure S4: ^1^H NMR of (*R*)-2-((1*R*,2*R*,4*R*)-2-(benzyloxy)-4-methylcyclohexyl)-2-methyloxirane (A); Figure S5: ^13^C NMR of (*R*)-2-((1*R*,2*R*,4*R*)-2-(benzyloxy)-4-methylcyclohexyl)-2-methyloxirane (A); Figure S6: ^1^H-^1^H Cosy NMR spectrum of (*R*)-2-((1*R*,2*R*,4*R*)-2-(benzyloxy)-4-methylcyclohexyl)-2-methyloxirane (A); Figure S7: Noesy NMR spectrum of (*R*)-2-((1*R*,2*R*,4*R*)-2-(benzyloxy)-4-methylcyclohexyl)-2-methyloxirane (A); Figure S8: HMQC spectrum of (*R*)-2-((1*R*,2*R*,4*R*)-2-(benzyloxy)-4-methylcyclohexyl)-2-methyloxirane (A); Figure S9: HMBC spectrum of (*R*)-2-((1*R*,2*R*,4*R*)-2-(benzyloxy)-4-methylcyclohexyl)-2-methyloxirane (A); Figure S10: ^1^H NMR of (*S*)-2-((1*R*,2*R*,4*R*)-2-(benzyloxy)-4-methylcyclohexyl)-2-methyloxirane (B); Figure S11: ^13^C NMR of (*S*)-2-((1*R*,2*R*,4*R*)-2-(benzyloxy)-4-methylcyclohexyl)-2-methyloxirane (B); Figure S12: ^1^H-^1^H Cosy NMR spectrum of (*S*)-2-((1*R*,2*R*,4*R*)-2-(benzyloxy)-4-methylcyclohexyl)-2-methyloxirane (B); Figure S13: Noesy NMR spectrum of (*S*)-2-((1*R*,2*R*,4*R*)-2-(benzyloxy)-4-methylcyclohexyl)-2-methyloxirane (B); Figure S14: HMQC spectrum of (*S*)-2-((1*R*,2*R*,4*R*)-2-(benzyloxy)-4-methylcyclohexyl)-2-methyloxirane (B); Figure S15: HMBC spectrum of (*S*)-2-((1*R*,2*R*,4*R*)-2-(benzyloxy)-4-methylcyclohexyl)-2-methyloxirane (B).
