# Supplementary material for: Bioinspired-Metalloporphyrin Magnetic Nanocomposite as a Reusable Catalyst for Synthesis of Diastereomeric (−)-Isopulegol Epoxide: Anticancer Activity Against Human Osteosarcoma Cells (MG-63)
Source: Molecules. 2018 Dec 24;24(1):52. doi: 10.3390/molecules24010052 (PMC6337611; doi:10.3390/molecules24010052)
Supplement: Supplementary file 1 [file molecules-24-00052-s001.pdf]

Supporting Information

for

Bioinspired-Metalloporphyrin Magnetic Nanocomposite as a Reusable Catalyst for Synthesis of Diastereomeric (-)-isopulegol Epoxide. Anticancer Activity Against Human Osteosarcoma Cells (MG-63)

Lucas D. Dias [1,2], Ana L. M. Batista de Carvalho [2], Sara M. A. Pinto [1], Gilberto L. B. Aquino [1,3], Mário J. F. Calvete [1], Liane M. Rossi [4], M. P. M. Marques [2,5]* and Mariette M. Pereira [1,*]

[1] Coimbra Chemistry Centre, *CQC*, Departament of Chemistry, Faculdade de Ciências e Tecnologia da Universidade de Coimbra, Rua Larga, 3004-535 Coimbra, Portugal; lucasdanillodias@gmail.com (L.D.D.); smpinto@qui.uc.pt (S.M.A.P); mcalvete@qui.uc.pt (M.J.F.C.)
[2] Unidade de I&D Química-Física Molecular, Department of Chemistry, University of Coimbra, Rua Larga, 3004-535, Coimbra, Portugal; almbc@ci.uc.pt (A.L.M.B.C)
[3] Faculty of Pharmacy, State University of Goiás, Fazenda Barreira do Meio, 75132-400, Anápolis, GO, Brazil; gilberto.benedito@ueg.br (G.L.B.A);
[4] Departamento de Química Fundamental, Instituto de Química, Universidade de São Paulo, Av. Prof. Lineu Prestes, 748, 05508-000, São Paulo, Brazil; lrossi@iq.usp.br (L.M.R);
[5] Department of Life Sciences, University of Coimbra, Calçada Martim de Freitas, 3000-456, Coimbra, Portugal;
* Correspondence: pmc@ci.uc.pt (M.P.M.M); mmpereira@qui.uc.pt (M.M.P)
Tel.: +351-239-826-541 (M.P.M.M.); +351-239-854-474 (M.M.P.)

Characterization of (–)-isopulegol benzyl ether (1)

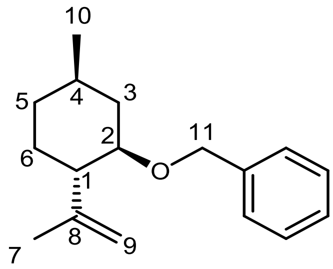

^{1}H-NMR ($CDCl_3$, 400 MHz) δ H (ppm): 7.32-7.19 (m, 5H, H*Ar*), 4.79-4.80 (m, 2H, H*9*), 4.59 (d, *J* = 16.0 Hz, 1H, H*11*), 4.41 (d, *J* = 16.0 Hz, 1H, H*11*), 3.28 (ddd, *J1* = 6.0 Hz, *J2* = 6.0 Hz, *J3* = 6.0 Hz, 1H, *H2*), 2.18-2.03 (m, 2H, C*H*), 1.68-1.67 (m, 3H, CH_3-*7*), 1.65-1.60 (m, 2H, C*H*), 1.41-1.25 (m, 2H, C*H*), 0.93 (s, *J* = 9.0 Hz, 3H, CH_3-*5*), 0.99-0.90 (m, 2H, C*H*).

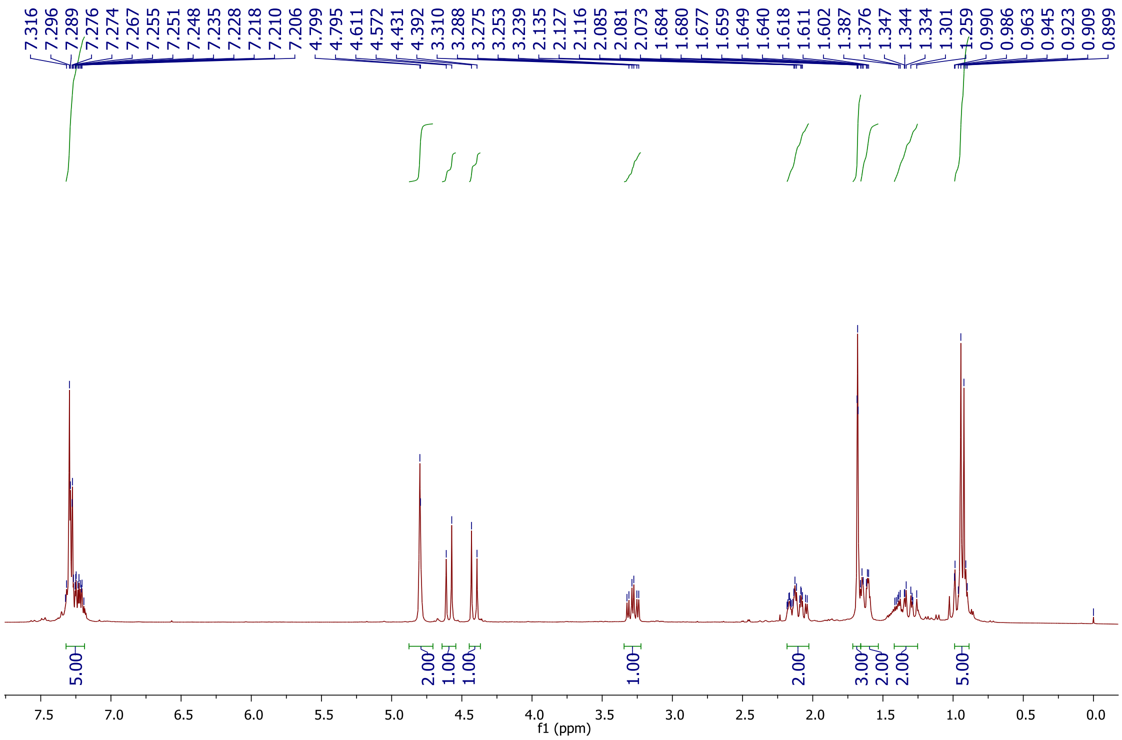

Figure S1. ^{1}H NMR spectrum of (–)-isopulegol benzyl ether (**1**).

^{13}C-NMR (101 MHz, CDCl$_3$) δ C (ppm): 20.1 (C-7), 22.20 (C-10), 31.1 (C-6), 31.6 (C-4), 34.5 (C-5), 40.3 (C-3), 51.8 (C-1), 70.4 (C-11), 79.2 (C-2), 111.1 (C-8), 127.3 ($C_{Ar.}$), 127.6 (C_{Ar}), 128.2 (C_{Ar}), 139.2 (C_{Ar}), 147.9 (C-9).

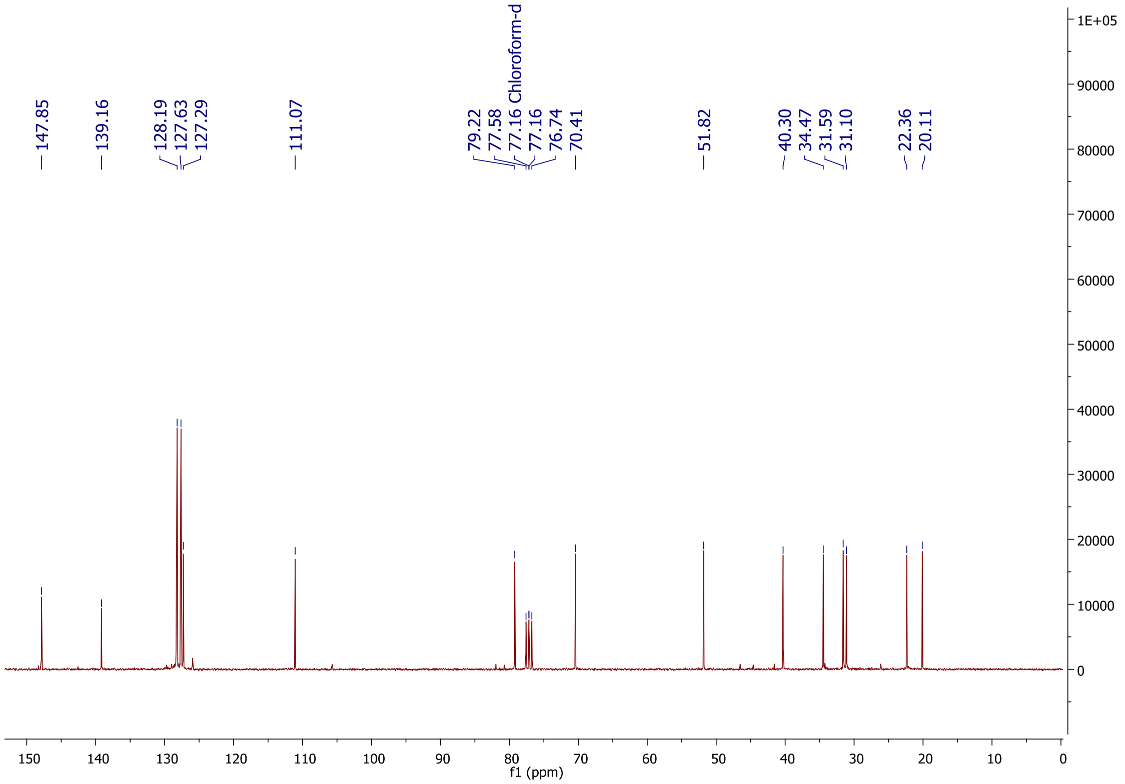

Figure S2. ^{13}C NMR spectrum of (–)-isopulegol benzyl ether (**1**).

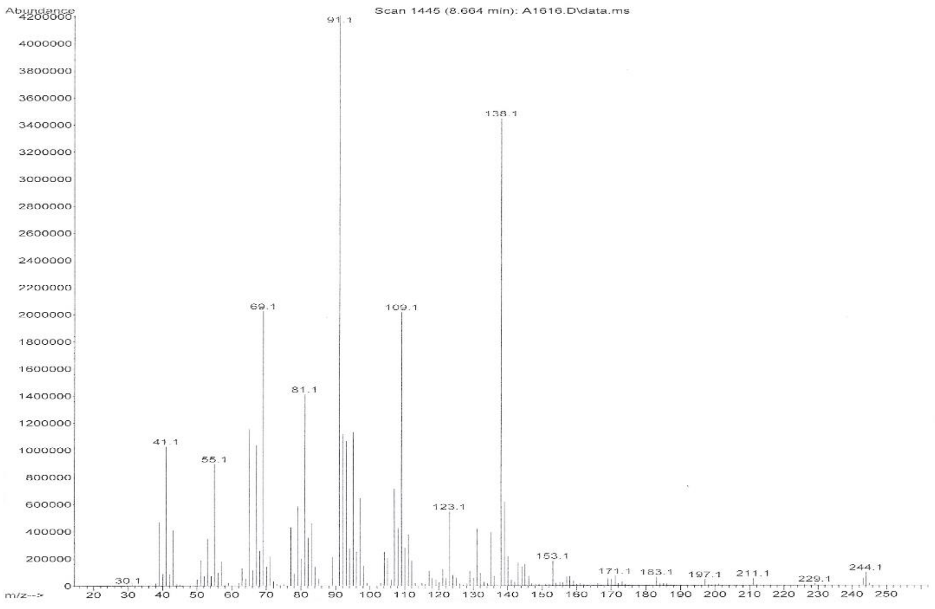

Figure S3. MS spectrum of (–)-isopulegol benzyl ether (**1**).

(-)-Isopulegol Benzyl Ether Epoxide –

(*R*)-2-((1*R*,2*R*,4*R*)-2-(benzyloxy)-4-methylcyclohexyl)-2-methyloxirane (**A**)

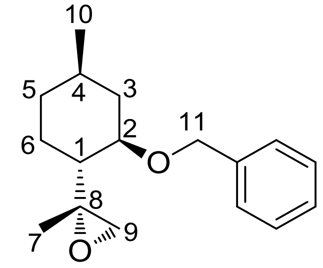

^{1}H-NMR ($CDCl_3$, 400 MHz) δ H (ppm): 7.36-7.25 (m, 5H, *HAr*), 4.68 (d, *J* = 12.0 Hz, 1H, *H11*), 4.40 (d, *J* = 12.0 Hz, 1H, *H11*), 3.26 (ddd, J_1 = 4.0 Hz, J_2 = 4.0 Hz, J_3 = 4.0 Hz, 1H, *H2*), 2.76 (d, *J* = 8.0 Hz, 1H, *H9*), 2.68 (d, *J* = 4.0 Hz, 1H, *H9*), 2.24-2.19 (m, 1H, *H3*), 1.90-1.86 (m, 1H, *H6*), 1.71-1.66 (m, 1H, *H5*), 1.43-1.39 (m, 1H, *H6*), 1.31-1.24 (m, 1H, *H4*), 1.13 (s, 3H, CH_3-*7*), 1.11-1.08 (m, 1H, *H1*), 0.95 (d, *J* = 8.0 Hz, 3H, CH_3-10), 0.92-0.86 (m, 2H, *H3*, *H5*).

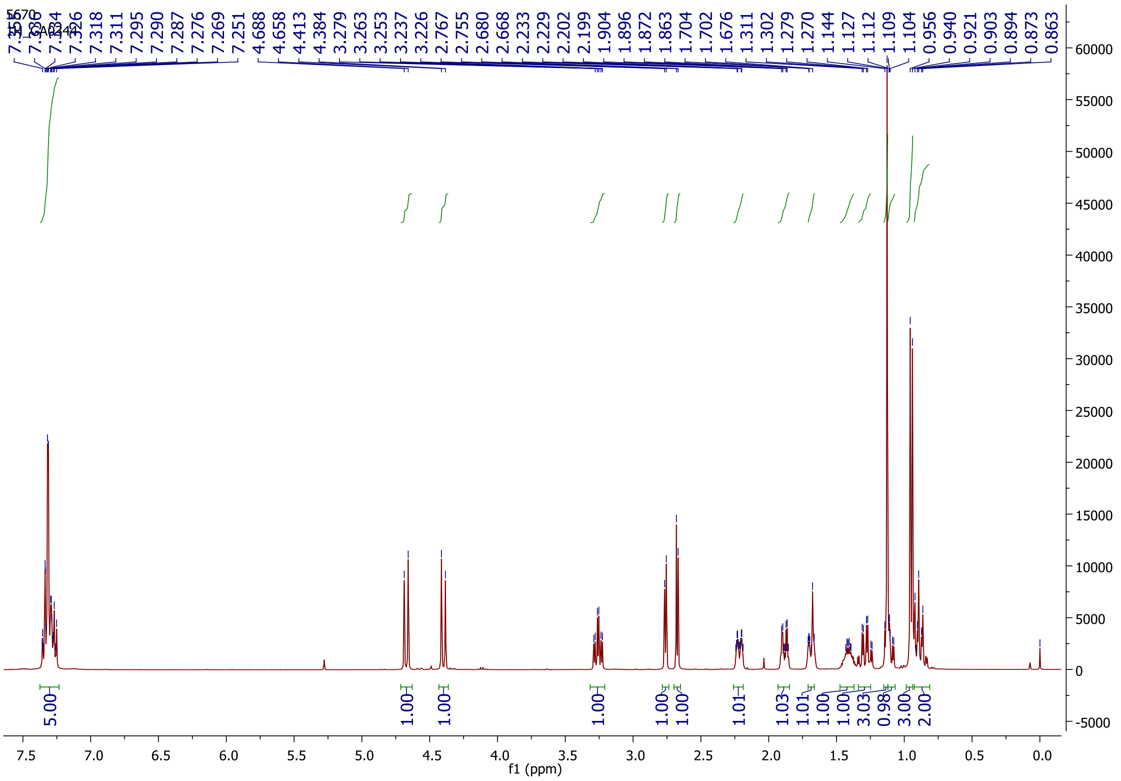

Figure S4. ^{1}H NMR of (*R*)-2-((1*R*,2*R*,4*R*)-2-(benzyloxy)-4-methylcyclohexyl)-2-methyloxirane (**A**).

^{13}C-NMR (101 MHz, $CDCl_3$) δ C (ppm): 16.7 (C-7), 22.3 (C-10), 26.4 (C-6), 31.5 (C-4), 33.9 (C-5), 39.9 (C-3), 50.8 (C-1), 56.8 (C-9), 57.9 (C-8), 70.2 (C-11), 78.8 (C-2), 127.6 (C-Ar), 128.4 (C-Ar), 139.0 (C-Ar).

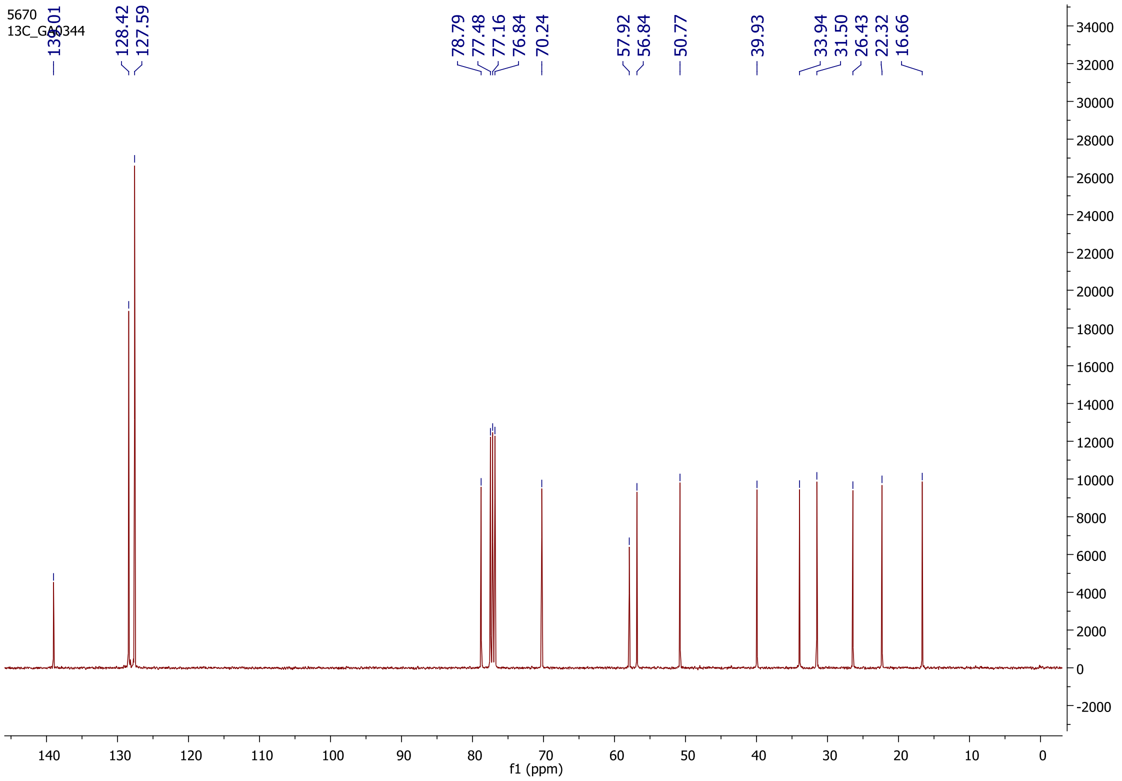

Figure S5. ^{13}C NMR of (*R*)-2-((1*R*,2*R*,4*R*)-2-(benzyloxy)-4-methylcyclohexyl)-2-methyloxirane (**A**).

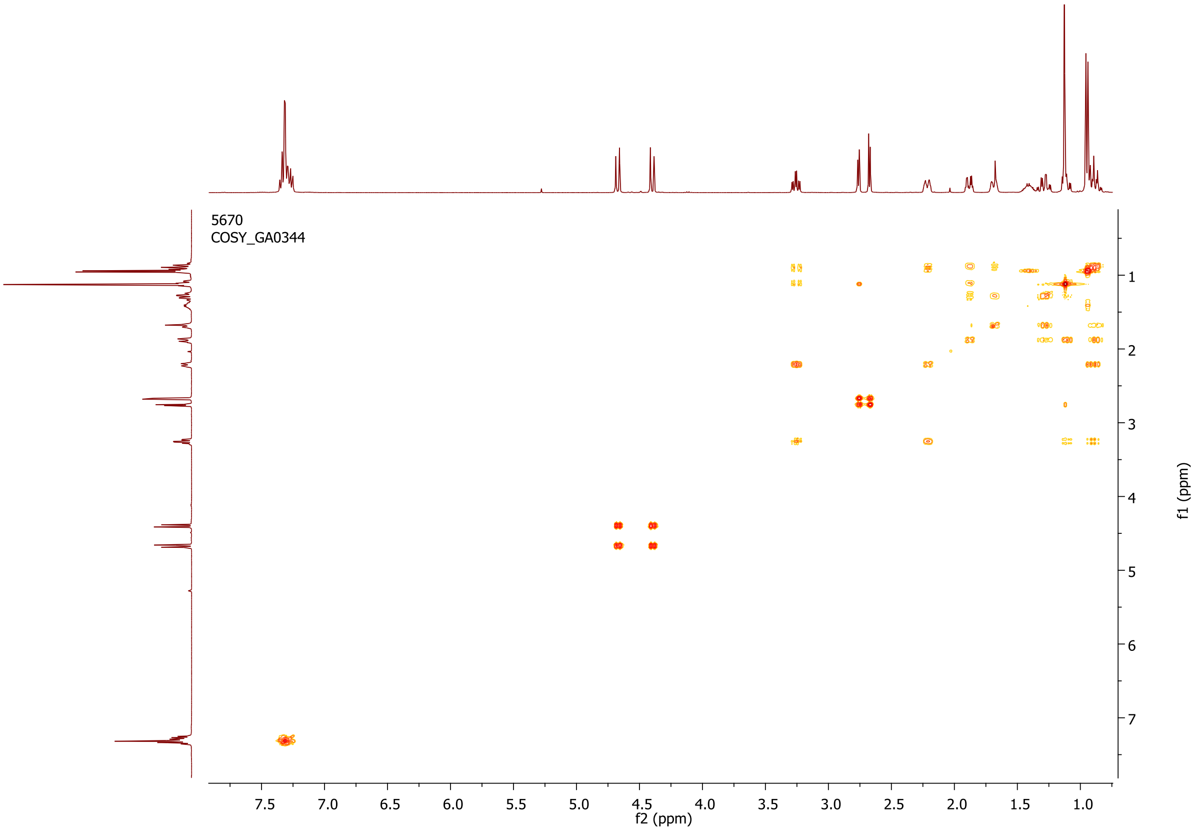

Figure S6. ^1H-^1H Cosy NMR spectrum of (*R*)-2-((1*R*,2*R*,4*R*)-2-(benzyloxy)-4-methylcyclohexyl)-2-methyloxirane (**A**).

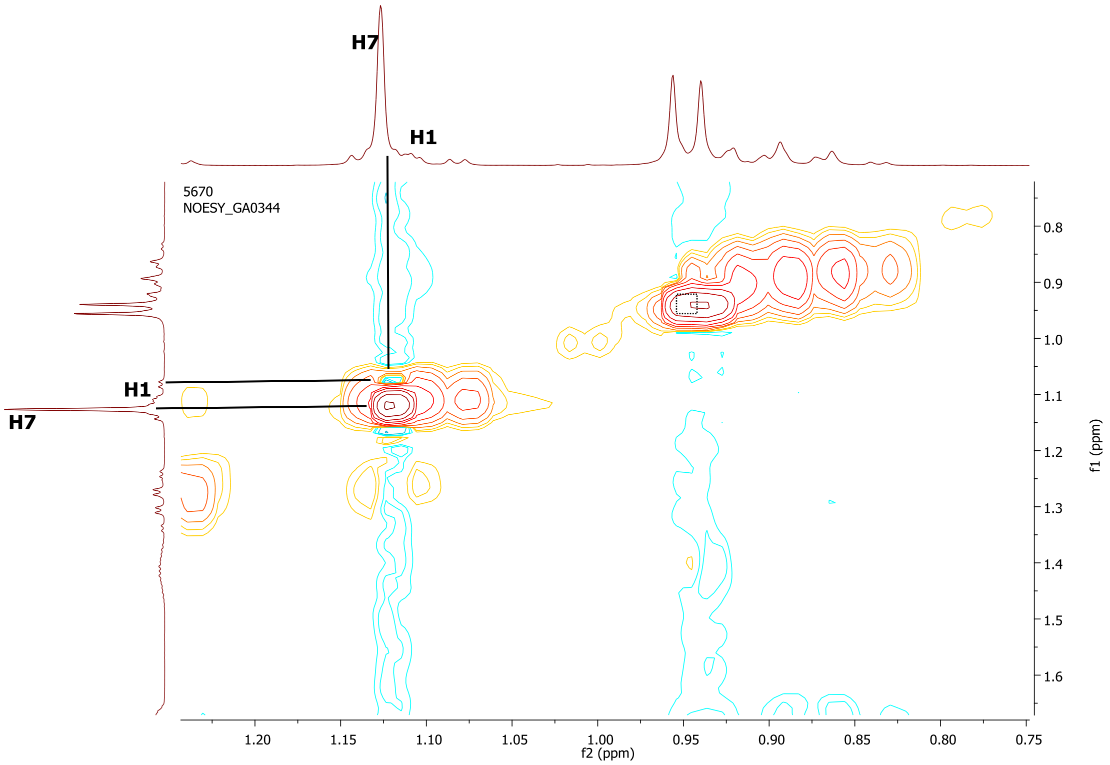

Figure S7. Noesy NMR spectrum of (*R*)-2-((1*R*,2*R*,4*R*)-2-(benzyloxy)-4-methylcyclohexyl)-2-methyloxirane (**A**).

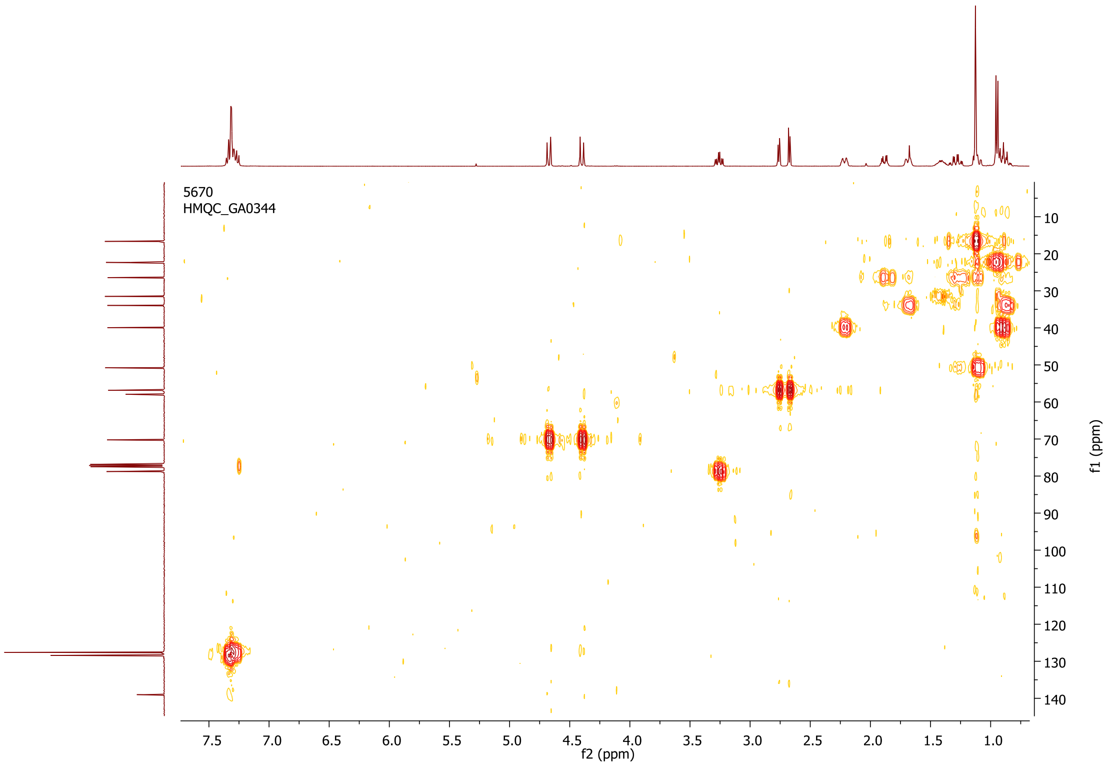

Figure S8. HMQC spectrum of (*R*)-2-((1*R*,2*R*,4*R*)-2-(benzyloxy)-4-methylcyclohexyl)-2-methyloxirane (**A**).

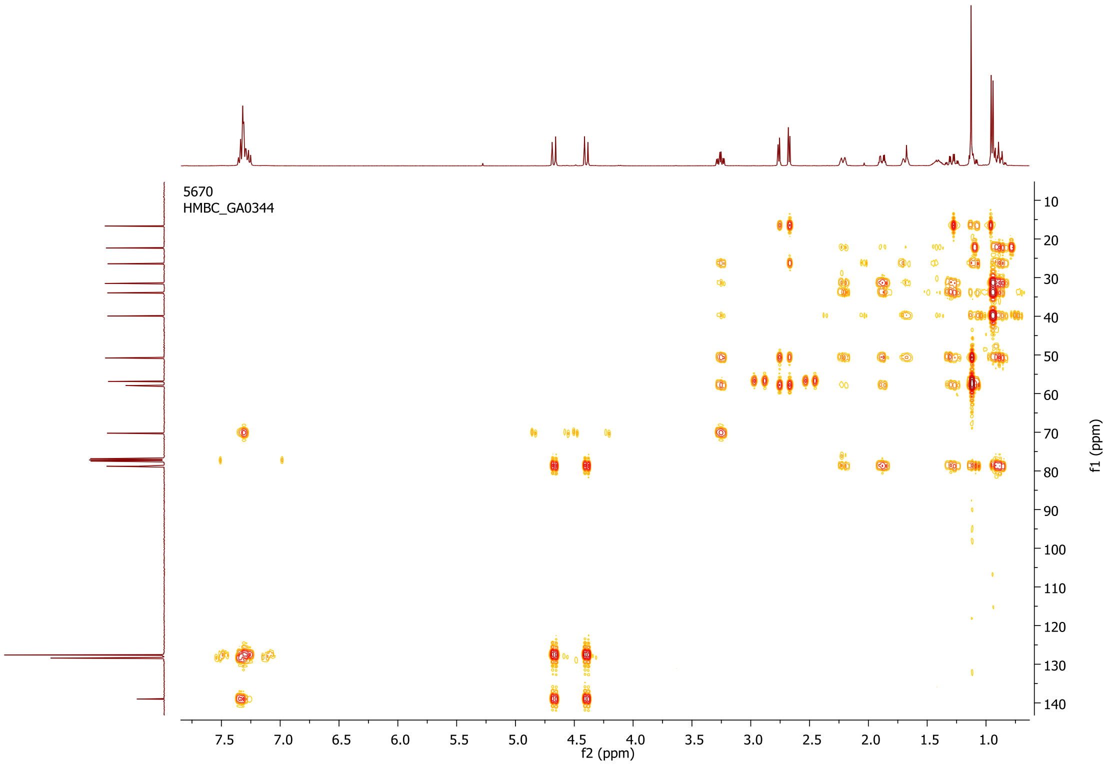

Figure S9. HMBC spectrum of (*R*)-2-((1*R*,2*R*,4*R*)-2-(benzyloxy)-4-methylcyclohexyl)-2-methyloxirane (**A**).

Characterization of (*S*)-2-((*1R*,*2R*,*4R*)-2-(benzyloxy)-4-methylcyclohexyl)-2-methyloxirane (**B**)

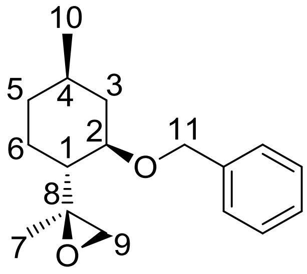

^{1}H-NMR (CDCl$_3$, 400 MHz) δ H (ppm): 7.42-7.40 (m, 2H, *HAr*), 7.35-7.31 (m, 2H, *HAr*), 7.26-7.23 (m, 1H, *HAr*), 4.72 (d, *J* = 12.0 Hz, 1H, *H11*), 4.44 (d, *J* = 12.0 Hz, 1H, *H11*), 3.32-3.26 (m, 1H, *H2*), 2.51-2.48 (m, 2H, *H9*), 2.23-2.20 (m, 1H, *H3*), 1.75-1.70 (m, 1H, *H6*), 1.69-1.64 (m, 1H, H5), 1.45-1.35 (m, 1H, *H4*), 1.34-1.30 (m, 1H, *H1*), 1.25 (s, 3H, *H7*), 1.21-1.11 (m, 1H, *H6*), 0.95 (d, *J* = 6Hz, 3H, *H10*), 0.92-0.85 (m, 2H, *H3* and *H5*).

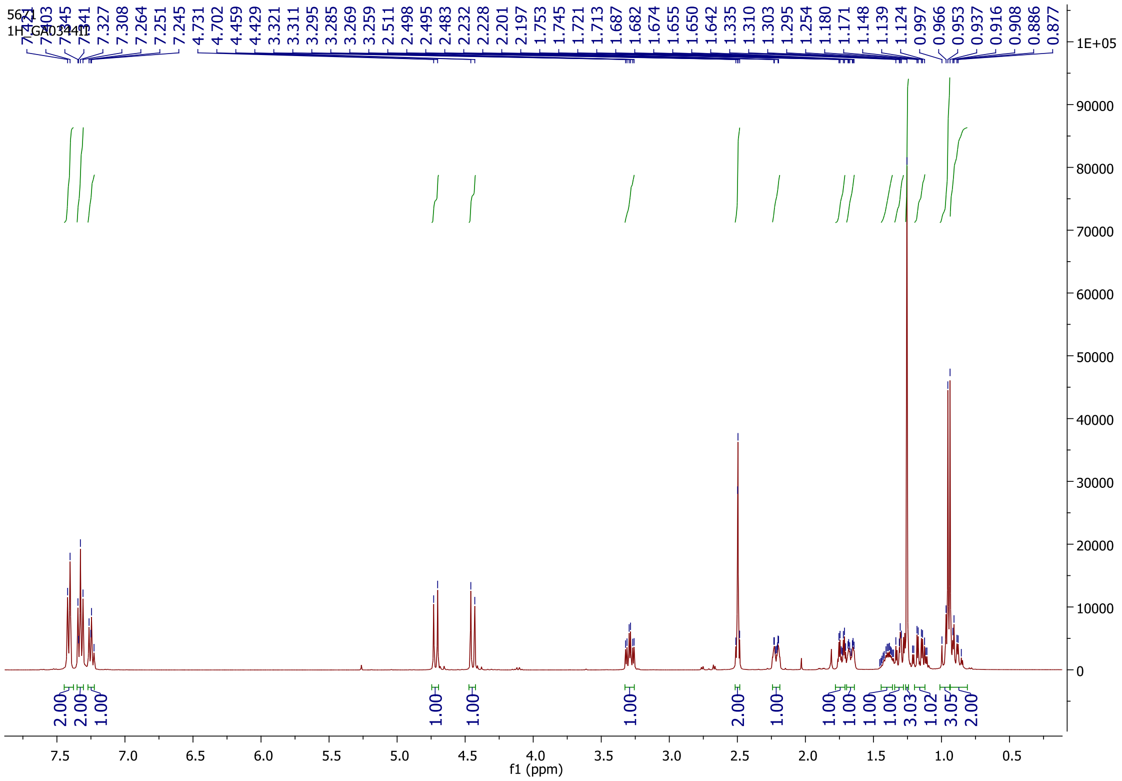

Figure S10. ^{1}H NMR of (*S*)-2-((*1R*,*2R*,*4R*)-2-(benzyloxy)-4-methylcyclohexyl)-2-methyloxirane (**B**).

^{13}C-NMR (101 MHz, CDCl$_3$) δ C (ppm): 18.9 (C-7), 22.2 (C-10), 27.8 (C-4), 31.3 (C-6), 34.2 (C-5), 39.6 (C-3), 49.2 (C-1), 52.0 (C-9), 58.3 (C-8), 69.7 (C-11), 78.3 (C-2), 127.4 (C-Ar), 127.7 (C-Ar), 128.3 (C-Ar), 138.9 (C-Ar).

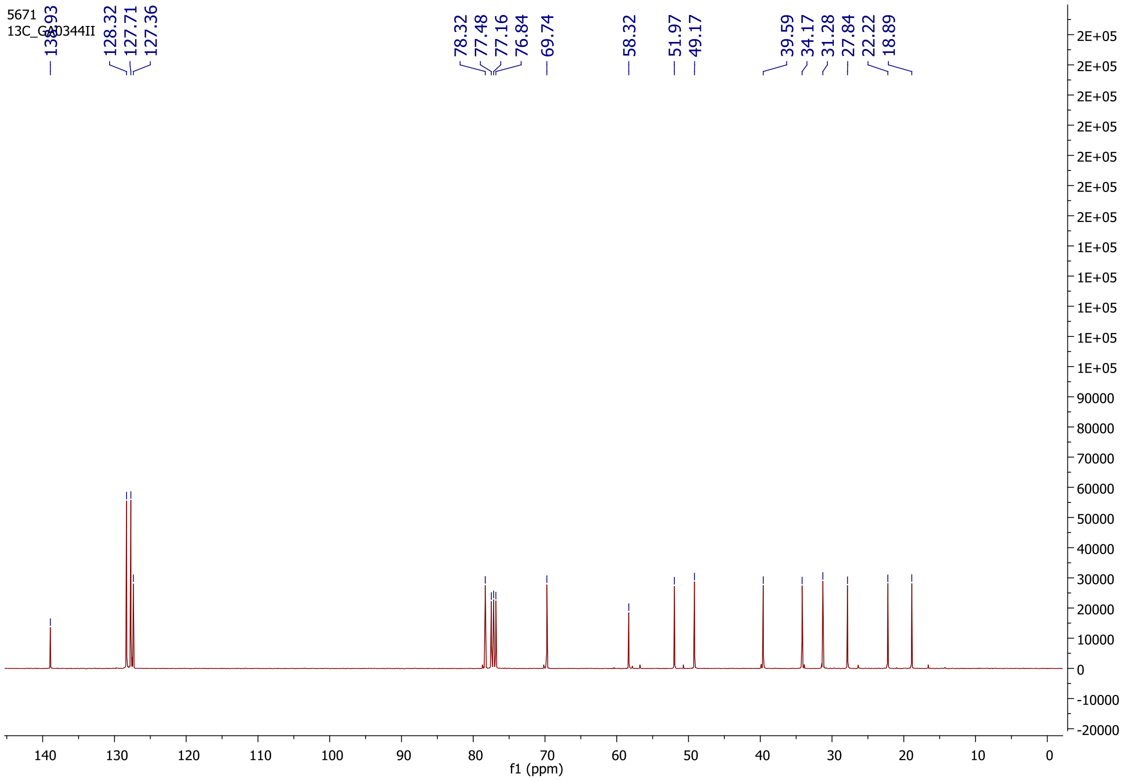

Figure S11. ^{13}C NMR of (*S*)-2-((*1R,2R,4R*)-2-(benzyloxy)-4-methylcyclohexyl)-2-methyloxirane (**B**).

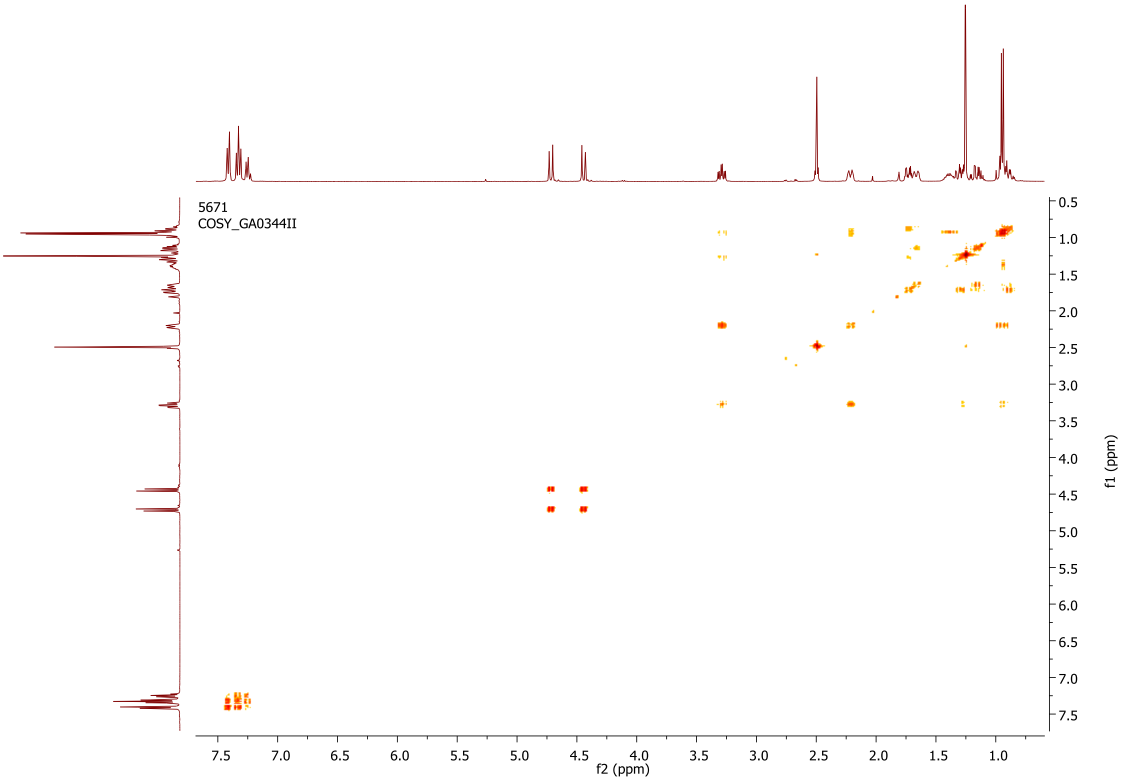

Figure S12. ^{1}H-^{1}H Cosy NMR spectrum of (*S*)-2-((*1R*,*2R*,*4R*)-2-(benzyloxy)-4-methylcyclohexyl)-2-methyloxirane (**B**).

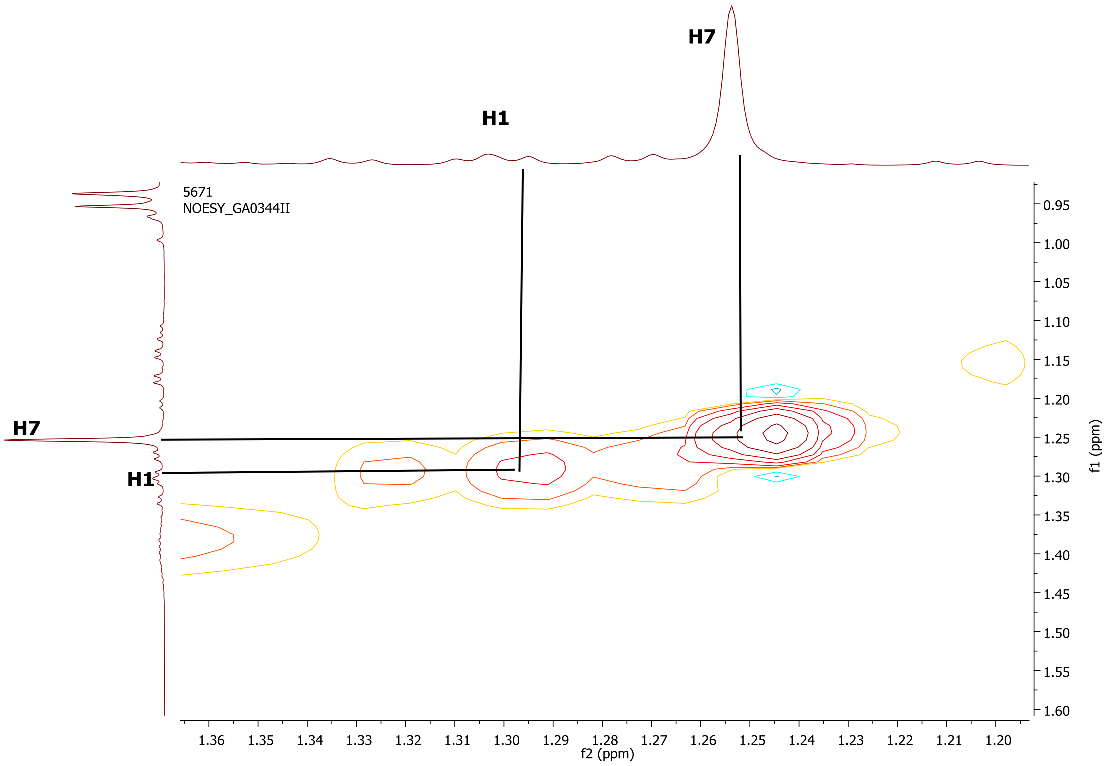

Figure S13. Noesy NMR spectrum of (*S*)-2-((*1R,2R,4R*)-2-(benzyloxy)-4-methylcyclohexyl)-2-methyloxirane (**B**).

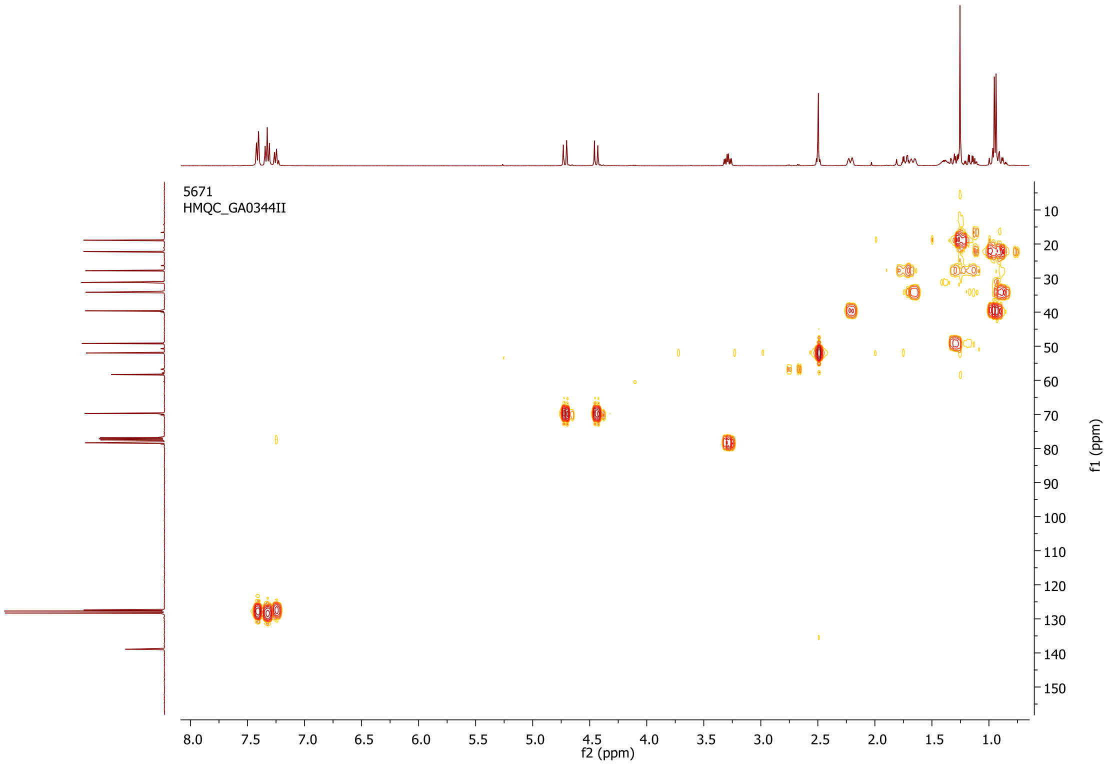

Figure S14. HMQC spectrum of (*S*)-2-((*1R*,*2R*,*4R*)-2-(benzyloxy)-4-methylcyclohexyl)-2-methyloxirane (**B**).

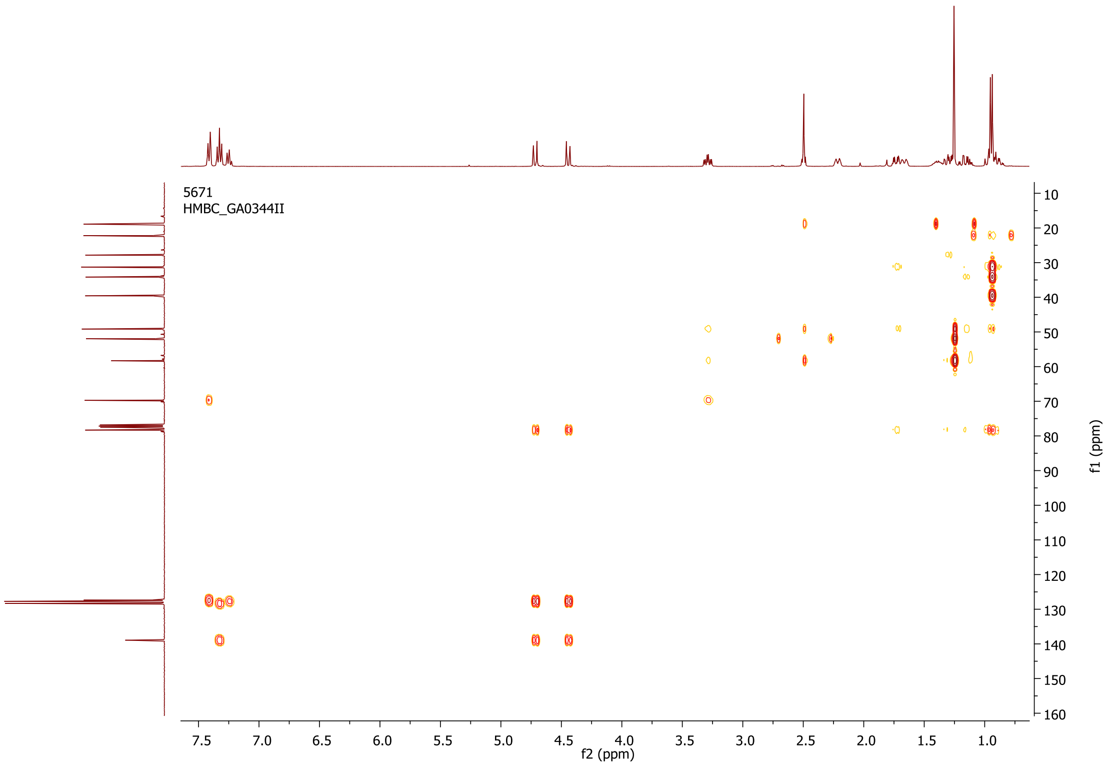

Figure S15. HMBC spectrum of (*S*)-2-((*1R*,*2R*,*4R*)-2-(benzyloxy)-4-methylcyclohexyl)-2-methyloxirane (**B**).
